# Patient satisfaction and complication rates in ultrasound-guided procedures: A questionnaire-based analysis

**DOI:** 10.6026/973206300221929

**Published:** 2026-04-30

**Authors:** Bhagya Sree Velamala, Zainab Haider Khan, R.A Siddiqui, Aisha Mohammed Kutty

**Affiliations:** 1 Department of Acute Medicine, Betsi Cadwaldr University Health Board, Wales, United Kingdom; 2 Department of Medicine, Avalon University School of Medicine, Willemstad CuraÇao; 3 Department of Pharmacology, NKP Salve Institute of Medical Sciences & RC and LMH, Nagpur, India; 4 Department of Oncology and Haematology, Portsmouth University Hospital NHS trust, Wessex, United Kingdom

**Keywords:** Interventional ultrasonography, patient-reported outcome measures (PROMs), clinical quality assessment, ultrasound-guided procedures (USGP)

## Abstract

Although ultrasound-guided procedures (USGP) are widely adopted to reduce technical complications, the relationship between patient satisfaction and procedural safety remains insufficiently explored. Most studies emphasise technical success while under-evaluating patient-reported outcomes as potential indicators of safety performance. Hence, this cross-sectional study assessed satisfaction among 300 adults undergoing USGP and examined its association with documented complication rates. A structured questionnaire evaluated comfort, communication, perceived safety and willingness for repeat procedures using a 10-point Likert scale. Among 270 respondents (90% response rate), the mean satisfaction score was 8.2 ± 1.1. Overall complication incidence was 2.2%, predominantly minor events. Patients with high satisfaction (≥9/10) demonstrated significantly lower complication rates compared with low satisfaction groups (0.7% versus 6.3%, p = 0.03). Satisfaction score independently predicted complication likelihood after adjustment for age and procedural complexity (β = -0.21, p = 0.01). Thus, we show that patient satisfaction may function as an adjunct safety indicator rather than mere experiential metric. Incorporating structured satisfaction assessment into procedural quality frameworks may enhance both patient-centred care and safety monitoring.

## Background:

The increasing use of ultrasound-guided procedures (USGP) in multiple medical specialties is due to the real-time images obtained during the procedure, which improve the accuracy and safety of these procedures [1]. There is recent evidence to support that USGP techniques decrease technical failure and complication rates than landmark-based techniques [2]. The complication rates associated with USGP have historically been reported as low (≤ 3%), which further supports the use of USGP as the best practice in many interventional settings [3]. The measurement of patient-centred outcomes is now considered an essential aspect of healthcare quality [5]. Patient satisfaction is an overall indicator of comfort during procedures, quality of care provided by the healthcare professional, perceived safety of the procedure and the level of trust in the clinician [2, 6]. Patient satisfaction has also been associated with improved adherence to follow-up appointments, reduced anxiety during procedures and improved cooperation during procedures [3, 6]. Effective communication and providing reassurance during procedures have also been associated with the patient's experience during an ultrasound-guided procedure [4, 7]. While there has been some progress in this area, most of the existing literature on USGP has focused primarily on technical success and complication rates and has under-represented the importance of measuring patient-reported outcomes related to their experience during USGP [8].

New research has started to identify how a patient's experience and satisfaction may impact procedural safety through behavioural and psychologically based mechanisms such as increased levels of cooperation, decreased levels of movement and the earlier identification of discomfort [2, 3]. However, the extent to which satisfaction affects the complication rates of ultrasound-guided procedures is currently poorly understood. The relationship between patient satisfaction and complication risk is important for quality improvement, patient education and procedural governance. Combining the use of patient-reported measures of satisfaction with patient safety metrics will give a more accurate representation of the quality of ultrasound-guided procedures [6, 7, 8 - 9]. Therefore, it is of interest to measure and examine patient satisfaction to establish the relationship between patient satisfaction and complication rates following ultrasound-guided procedures.

##  Materials and Methods:

## Study design and setting:

This cross-sectional observational study was conducted at a tertiary care centre over six months. The study evaluated patient satisfaction following ultrasound-guided procedures and its association with early complications.

## Participants:

Adults aged ≥18 years that underwent ultrasound-guided procedures were eligible. Patients unable to provide informed consent or undergoing emergency interventions were excluded.

## Sample size and recruitment:

Three hundred consecutive patients were invited to participate. All participants provided written informed consent prior to enrolment.

## Satisfaction assessment:

A validated questionnaire was administered immediately after the procedure. The survey evaluated four domains: procedural comfort, communication and explanation, perceived safety and willingness to repeat the procedure. Responses were recorded using a 10-point Likert scale.

Satisfaction scores were categorised as:

[1] Low: <7

[2] Moderate: 7-8

[3] High: ≥9

## Clinical and procedural data collection:

Demographic variables included age and sex. Procedural data included type and complexity of ultrasound-guided intervention.

## Complication assessment:

Institutional records were reviewed for complications occurring within 24 hours post-procedure. Complications were classified as minor or major. Events were matched to questionnaire responses using anonymized patient identifiers.

## Statistical analysis:

Statistical analysis was performed using SPSS version 26.0. Descriptive statistics summarised demographic and procedural characteristics. Chi-square tests compared complication incidence across satisfaction categories. Independent t-tests compared mean satisfaction scores between patients with and without complications. Pearson correlation assessed the relationship between continuous satisfaction scores and complication occurrence. Multivariate logistic regression evaluated satisfaction as an independent predictor of complications after adjustment for age, sex and procedural complexity.

## Ethical approval:

The study received institutional review board approval. All participants provided written informed consent.

## Results:

A total of 300 patients were invited to participate and 270 completed the survey, giving a response rate of 90%. The study population had a mean age of 52.4 ± 14.5 years and females comprised 58% of participants. Ultrasound-guided vascular access was the most frequently performed procedure, followed by biopsy, nerve block and fluid aspiration. Overall satisfaction scores were high, with a mean score of 8.2 ± 1.1. Most patients reported high satisfaction (58.1%), while only 11.9% reported low satisfaction. Satisfaction was consistently high across all assessed domains, particularly communication and willingness to repeat the procedure. The overall complication rate was 2.2% (6/270) and most complications were minor events such as localized hematoma or transient pain. Complication incidence decreased progressively across satisfaction categories, demonstrating a gradient association. Patients with high satisfaction experienced the lowest complication rates, whereas the low satisfaction group demonstrated the highest incidence. Correlation analysis showed a modest negative association between satisfaction score and complication occurrence (r = -0.24). Age showed minimal correlation with complications, while procedural complexity demonstrated a weak positive relationship. Multivariate regression confirmed satisfaction score as an independent predictor of complication likelihood after adjusting for age, sex and procedure complexity. Detailed demographic characteristics, satisfaction distributions, complication rates, correlation analysis and regression results are presented in Tables 1-6. (see PDF) Table 1 (see PDF) describes the demographic profile indicates a middle-aged cohort with female predominance, reflecting the typical population undergoing elective ultrasound-guided procedures. The procedural distribution shows that vascular access and biopsies represent the majority of indications, suggesting that findings are most applicable to commonly performed interventional ultrasound settings. The diversity of procedures also supports the generalizability of satisfaction assessment across different intervention types. Table 2 (see PDF) shows the strong predominance of high satisfaction (58.1%) indicates that ultrasound-guided procedures are generally well accepted by patients. The relatively small low-satisfaction group (11.9%) suggests that negative experiences are uncommon but clinically relevant for quality improvement efforts. Table 3 (see PDF) depicts communication and willingness to repeat the procedure achieved the highest satisfaction scores. This pattern suggests that patient-clinician interaction and perceived reassurance are central drivers of overall satisfaction. Slightly lower scores for perceived safety indicate that reassurance about procedural safety may still be improved. Table 4 (see PDF) shows a clear gradient relationship exists between satisfaction level and complication incidence. Patients with low satisfaction experienced the highest complication rates, while highly satisfied patients experienced the lowest rates. This pattern supports a meaningful association between patient experience and procedural safety. Table 5 (see PDF) presents the negative correlation between satisfaction and complication occurrence indicates that higher satisfaction is associated with fewer complications. The weak correlation between age and complications suggests that patient experience may be more relevant than demographic factors. The positive correlation between procedural complexity and complications is expected and supports the validity of the dataset. Table 6 (see PDF) shows multivariate analysis confirms that satisfaction independently predicts complication likelihood even after adjustment for confounders. This finding suggests that patient satisfaction is not merely a reflection of procedure complexity or demographic characteristics. Instead, satisfaction appears to represent an independent dimension related to procedural safety and care quality.

## Discussion:

This study evaluated patient satisfaction following ultrasound-guided procedures and examined its association with early complication rates. The findings demonstrate consistently high satisfaction across multiple domains and identify a significant inverse relationship between satisfaction scores and complication occurrence. Satisfaction remained an independent predictor of complications after adjusting for age and procedural complexity. These results support the growing recognition that patient-reported experience measures can complement traditional safety metrics in interventional practice [10]. Ultrasound guidance has transformed procedural medicine by enabling real-time visualization, improved accuracy and reduced technical complications. Contemporary evidence shows that ultrasound-guided interventions are associated with lower complication rates and improved procedural success across vascular access, musculoskeletal interventions and regional anaesthesia [11, 12]. The low complication rate observed in this study is consistent with the established safety profile of ultrasound-guided techniques in routine clinical practice. A notable finding is the graded association between satisfaction and complications. Patients reporting high satisfaction experienced the lowest complication incidence, whereas those with low satisfaction demonstrated the highest rates [13]. This pattern suggests that patient experience may influence procedural safety through behavioural and communication-related pathways. Effective communication and reassurance reduce anxiety and improve cooperation during procedures, which may reduce movement, facilitate accurate needle placement and enable earlier reporting of discomfort [2, 3]. These factors can contribute to reduced minor adverse events. Domain-specific findings further highlight the central role of communication and patient engagement. Communication and willingness to repeat the procedure achieved the highest satisfaction scores [14].

Prior studies have shown that clear explanations and real-time reassurance improve patient experience during ultrasound-guided interventions [5, 6]. These observations emphasise that procedural quality extends beyond technical success to include interpersonal and communication skills. Multivariate analysis demonstrated that satisfaction remained independently associated with complication likelihood after adjusting for confounders [15]. This finding suggests that satisfaction reflects an additional dimension of care quality not captured by conventional clinical variables. Integrating patient satisfaction into routine procedural evaluation may therefore provide a more comprehensive assessment of safety and performance. From a clinical perspective, structured pre-procedural counselling, clear intra-procedural communication and post-procedural follow-up may improve both patient experience and safety outcomes [3, 7]. Incorporating patient-reported outcome measures into quality assurance programmes may help identify improvement opportunities that are not evident from complication audits alone. These findings extend current literature by positioning patient satisfaction as a measurable component of procedural safety and quality rather than solely an experiential outcome. This study has several limitations. The cross- sectional design prevents causal inference. The single-centre setting may limit generalisability. The low number of complications restricted evaluation of rare major events. Satisfaction was measured immediately after the procedure and may not reflect longer-term perspectives. Future research should include multicentre and longitudinal studies to further clarify the mechanisms linking patient experience and procedural safety.

## Conclusion:

Higher patient satisfaction with ultrasound-guided procedures is significantly associated with lower complication rates and independently predicts procedural safety. Data support the integration of structured patient-reported experience measures into routine quality and safety frameworks for ultrasound-guided interventions. Thus, strengthening communication, counselling and patient engagement may simultaneously improve patient experience and procedural outcomes.

## Acknowledgement:

lWe acknowledge that the first and second author contributed equally to this paper and hence they are considered as joint first author.
